# Supplementary figures and images for: The causal effect of obesity on prediabetes and insulin resistance reveals the important role of adipose tissue in insulin resistance
Source: PLoS Genet. 2020 Sep 14;16(9):e1009018. doi: 10.1371/journal.pgen.1009018 (PMC7515203; doi:10.1371/journal.pgen.1009018)

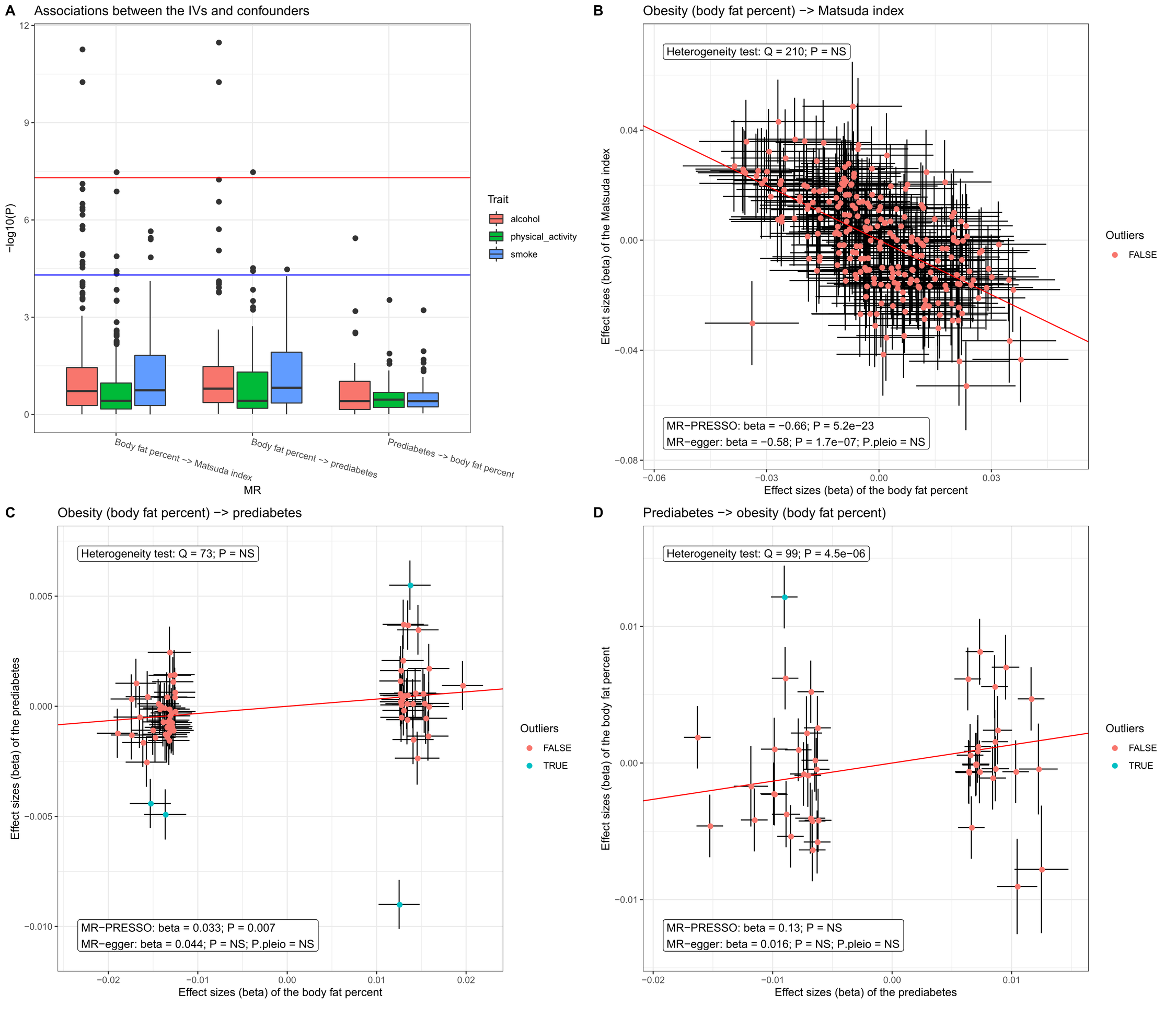

Supplement: S1 Fig — (A) The associations between all IVs and three potential confounders, i.e. alcohol intake, physical activity, and smoking. The x axis shows the MR analysis in which the IVs were used. The y axis shows the p-values of the association between the IVs and confounders. (B) The MR results of body fat percent on Matsuda index without using the confounder-associated variants as IVs. (C) The MR results of body fat percent on prediabetes without using the confounder-associated variants as IVs. (D) The MR results of prediabetes on body fat percent remained the same as in Fig 1D as none of the prediabetes IV SNPs were associated with the 3 tested confounders. (TIF) [file pgen.1009018.s001.tif]

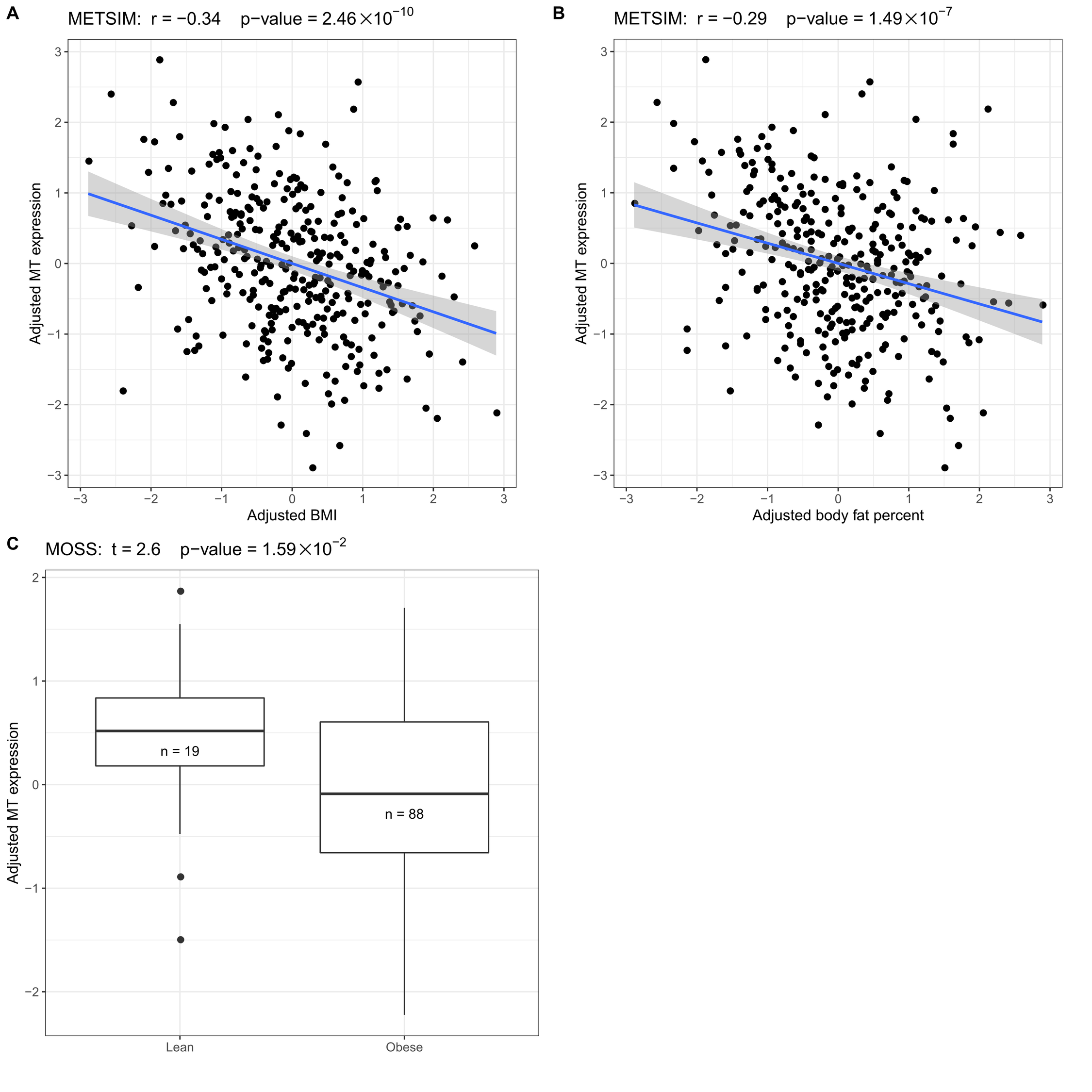

Supplement: S2 Fig — (A) BMI is significantly associated with the adjusted MT expression in a Pearson correlation test. (B) Body fat percent is significantly associated with the adjusted MT expression in a Pearson correlation test. (C) In MOSS, obese Mexicans have a lower adjusted MT expression when compared to the lean Mexicans. (TIF) [file pgen.1009018.s002.tif]

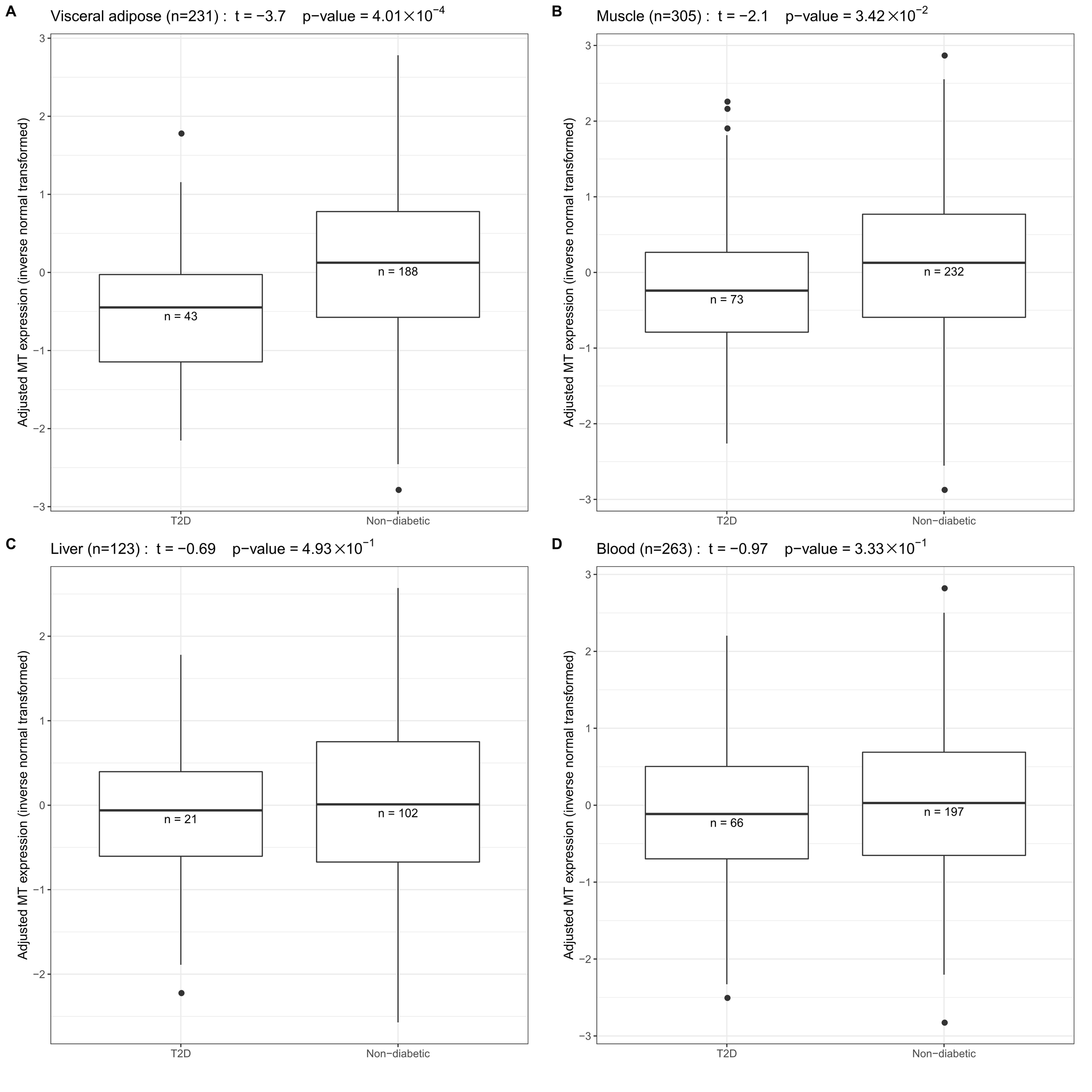

Supplement: S3 Fig — In (A) visceral adipose and (B) muscle, the adjusted MT expression is significantly higher in the non-diabetic individuals than in the T2D patients. In (C) liver and (D) blood, there is no evidence of differential MT gene expression between the non-diabetic individuals and the T2D patients. (TIF) [file pgen.1009018.s003.tif]

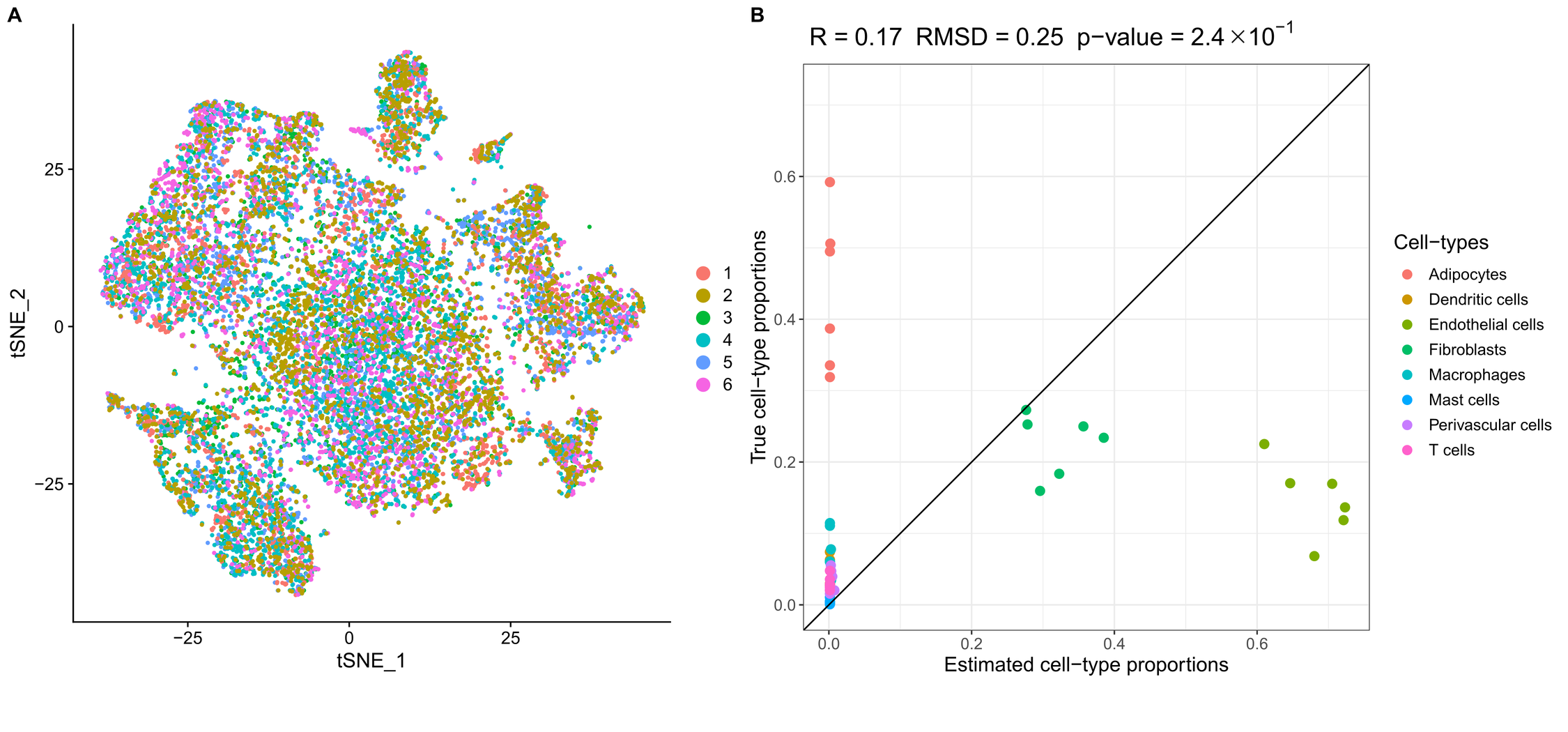

Supplement: S4 Fig — (A) The t-SNE plot shows no evidence of a batch effect in SN-RNA-seq clustering. The dots are colored by sample IDs. (B) When using all genes in the SN-RNA-seq data without any filtering, the estimated cell-type proportions are not concordant with the true cell-type proportions. Thus, using the selected genes (Fig 2B) performs much better than using all genes as reference in the decomposition process. (TIF) [file pgen.1009018.s004.tif]

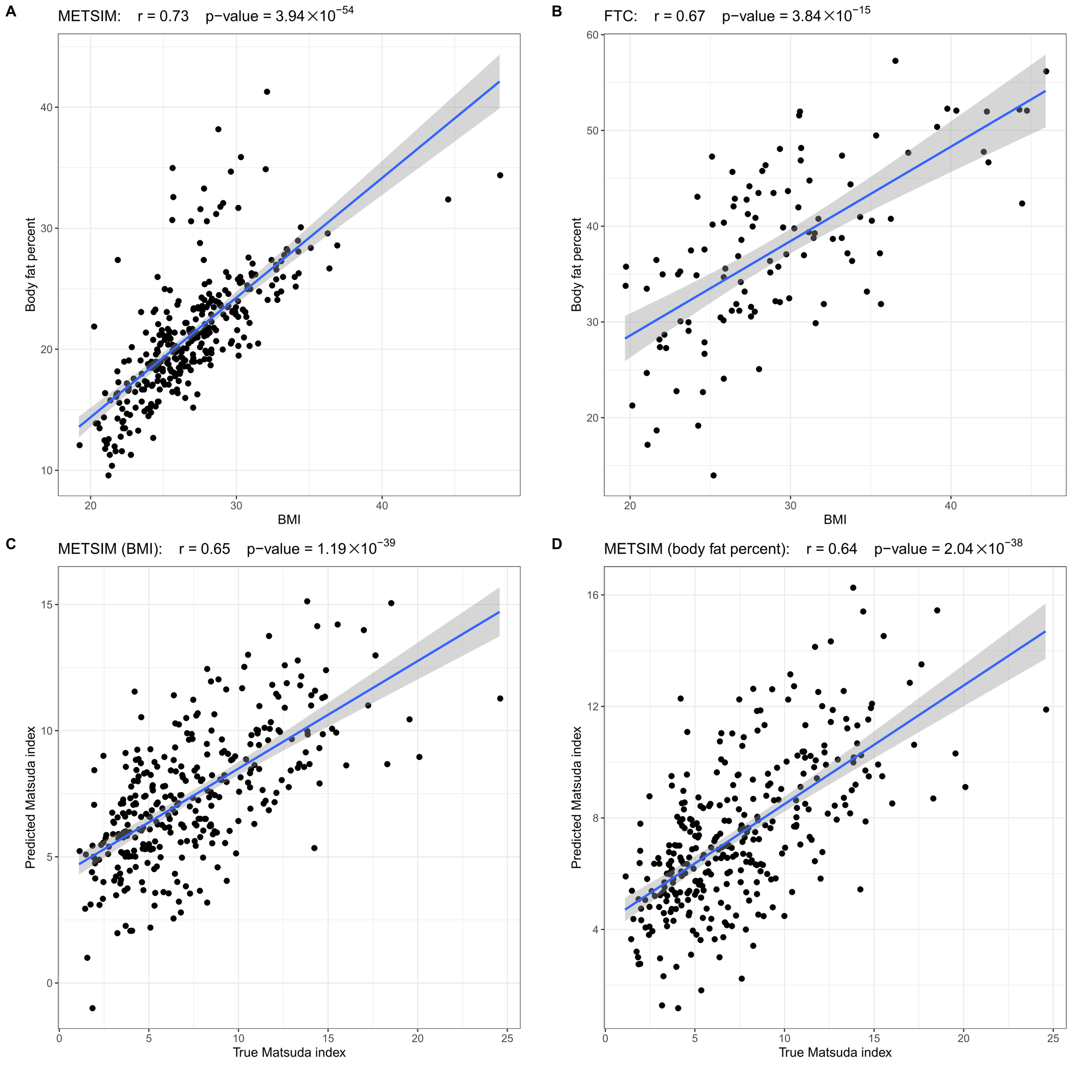

Supplement: S5 Fig — Using either BMI or body fat percent predicted Matsuda index in METSIM cohort in a similar accuracy. (A) The association between the body fat percent and BMI in METSIM. (B) The association between the body fat percent and BMI in FTC. (C) The association between the predicted Matsuda index and true Matsuda index when using BMI to represent obesity status. (D) The association between the predicted Matsuda index and true Matsuda index when using body fat percent to represent obesity status. (TIF) [file pgen.1009018.s005.tif]

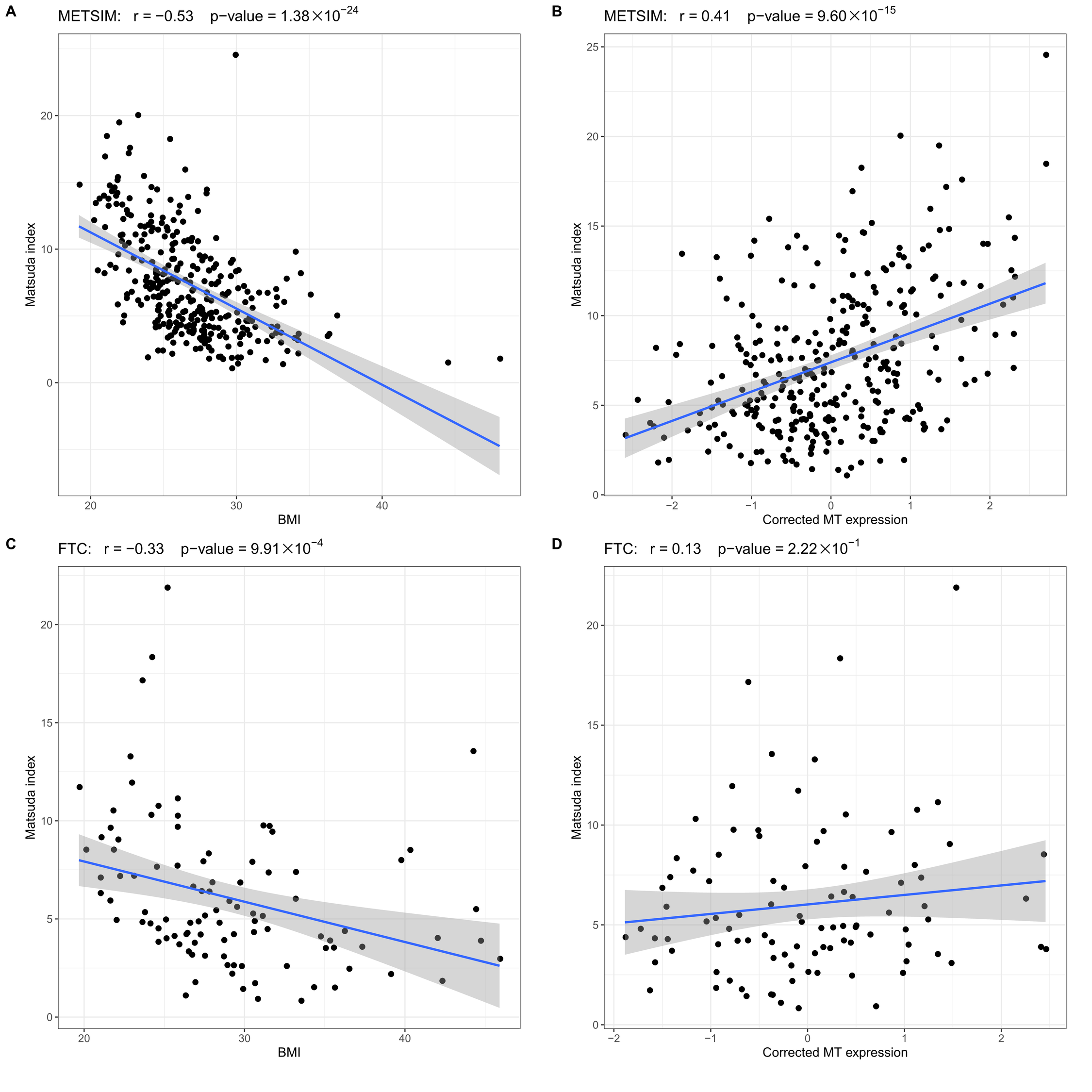

Supplement: S6 Fig — The predicted Matsuda index is always more strongly associated with the true Matsuda index than any of the predictors (Fig 4). (A) The correlation between raw BMI and the Matsuda index in METSIM. (B) The correlation between the corrected MT gene expression and Matsuda index in METSIM. (C) The correlation between raw BMI and the Matsuda index in FTC. (D) The correlation between the corrected MT gene expression and Matsuda index in FTC. (TIF) [file pgen.1009018.s006.tif]

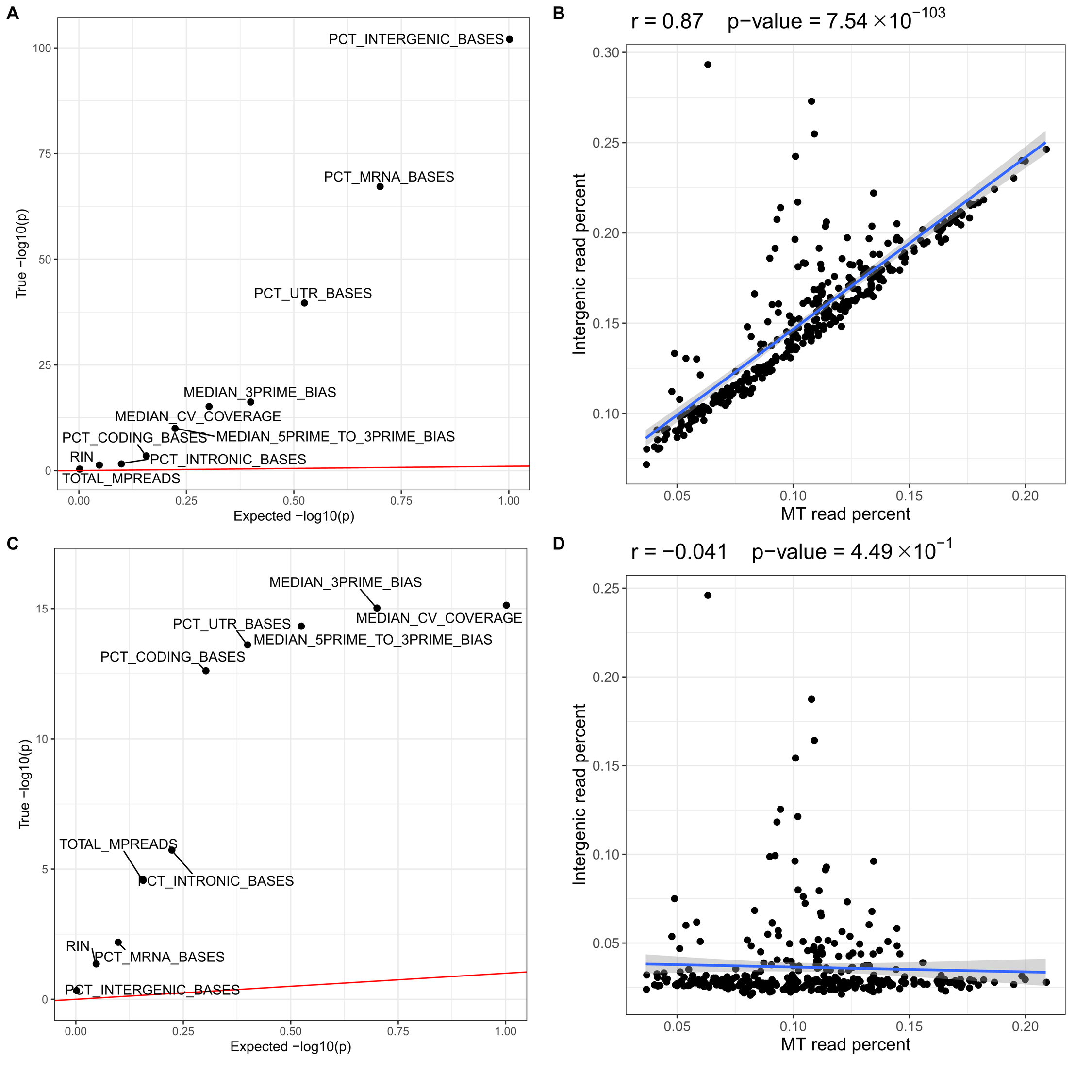

Supplement: S7 Fig — When excluding the MT reads to estimate the RNA metrics, the correlations between the MT read percent and other RNA metrics are reduced. (A). The qq-plot shows the correlations between the MT read percent and estimated RNA metrics, including MT reads. The y axis shows the -log10(p-value) of the associations between MT read percent and the technical factors estimated from the RNA-seq data. The x axis shows the expected -log10(p-value) if no true associations between the MT reads percent and technical factors exist. (B). The intergenic read percent is dominated by the MT read percent, including the MT reads. (C). The qq-plot shows the correlations between the MT read percent and estimated RNA metrics, excluding the MT reads. Compared to A, the associations between the MT read percent and other technical factors are much weaker. (D). The intergenic read percent is not correlated with the MT read percent, excluding the MT reads. (TIF) [file pgen.1009018.s007.tif]
