# Supplementary material for: The causal effect of obesity on prediabetes and insulin resistance reveals the important role of adipose tissue in insulin resistance
Source: PLoS Genet. 2020 Sep 14;16(9):e1009018. doi: 10.1371/journal.pgen.1009018 (PMC7515203; doi:10.1371/journal.pgen.1009018)
Supplement: Attachment — Submitted filename: Comments_LingyanCHEN.pdf [file pgen.1009018.s019.pdf]

General comments

In this manuscript, the authors describe the identification of causal effect of obesity on prediabetes and investigate the potential mechanisms via both bulk RNA sequencing and single nucleus RNA sequencing and find that individuals' BMI, adipose cell-type composition, and adipose MT gene expression predict their insulin resistance well.

I have three comments concerning the methods described in the overall well-written manuscript:

1) For the Mendelian randomization (MR) method reported in the manuscript, the author only used MR-PRESSO for both causal estimate and pleiotropy estimation. This method is quite powerful, however, more sensitivity analyses, e.g. heterogeneity test, should be performed to validate the robustness of the results. As this is the core assumption for all the follow-up experiments.
2) For the overall the statistical methods applied, there is no definition for the statistical significant threshold used. However, this is required to understand the potential type I errors with subsequent effects on power.
3) For the application of using adipose RNA-seq data to predict insulin resistance, the authors argue that the Matsuda Index is not available in many human metabolic cohorts, what about the RNA-seq data? Also, using RNA-seq data to predict the cell proportion and then for the prediction of Matsuda Index introduce noise from both steps.

Specific comments

MR analysis:

1) What are the exact SNPs used as IVs for BMI which cohort? Please provide the summary statistics of these SNPs.
2) Please provide MR sensitivity analysis to validate the causal estimates.
3) Hundreds of IVs were selected for BMI in UKBB, please provide the heterogeneity test statistics, e.g. Q stats.
4) Risk factors of prediabetes, such as blood pressure and dyslipidemia, may confound the causal effect of BMI -> prediabetes. The authors should remove instrumental variants (of BMI) that associated with these risk factors, otherwise it will violate the MR assumptions.
5) MR Steiger filtering can be used to estimate to causal effect direction if there is no available GWAS for Matsuda index.
6) For the interpretation of the MR results, please elaborate this on the results part.

Estimating adipose cell-type proportions and predicting insulin resistance:

1) Please provide the exact cell proportions for each of the 6 samples with sn-RNAseq data.

2) Please provide the detailed summary statistics (e.g. P values) for the correlation between estimated cell-type proportions and true proportion (Figure 3B)
3) Please provide the estimated cell proportions and s.e. if available, for 335 samples from the METSIM cohort.
4) From the multi-linear models, not all the factors included were significantly associated with the outcome (supp Table 3). However, in the prediction model (supp Table 4), all the variables were included. Note that the beta for "Mast cells" is 123.4385, this is highly suspicious. Please specify the criteria of variable selection for the prediction model as well as the model evaluation.

Figures & Figure legends:

1) There is no Figure legend for Figure 1C-D.
2) Please add the sample size from GTEx to Figure 2C & Figure 4B (box plots)
3) There is no Figure 3C in the Figure list
4) Please add detailed figure legend for Supp Figure 4A & 4C.
