# Supplementary material for: The causal effect of obesity on prediabetes and insulin resistance reveals the important role of adipose tissue in insulin resistance
Source: PLoS Genet. 2020 Sep 14;16(9):e1009018. doi: 10.1371/journal.pgen.1009018 (PMC7515203; doi:10.1371/journal.pgen.1009018)
Supplement: Attachment — Submitted filename: Reviewer2_0420ZM.docx [file pgen.1009018.s020.docx]

**Responses to Reviewer 2:**

We thank the Reviewer for the helpful critique and comments of the manuscript and have addressed all of the issues that were raised. We hope that the revisions satisfactorily respond to all of the Reviewer’s concerns. The changes are indicated in the marked-up copy of the revised manuscript.

**Reviewer #2: The aim of the study is to clarify the association between obesity and prediabetes using in part genetic epidemiological and in part cellular approaches. I was not fully comfortable with the way the study was conceptualised. There are also some issues with the methodology used for instrument selection and for the MR analyses in general. Comments below provide further details relating to my concerns.**

**Author summary was not conceptually clear, and it should be revised. It also illustrates some of the issues with the paper in general. Examples:**

**“Previous studies have shown that obesity has a causal effect on T2D;however, it remains unknown whether obesity causes prediabetes and insulin resistance already in non-diabetic individuals.”**

**I have a problem with logic underlying this conceptualisation, as if we know that obesity causes T2D, then obesity has to cause prediabetes in non-diabetic individuals before they progress to T2D. This is not to say that there could not be mechanistic insights we are still to gain in this context, or that elevated blood sugar would could not contribute in part to obesity, but overall I felt very underwhelmed by this conceptualisation.**

Response: We thank the Reviewer for this helpful comment. While we agree that prediabetes is an inevitable stage of T2D, we believe that our MR analyses contribute to the field as follows: 1) Not all of the people with prediabetes do not ultimately develop diabetes given that the annualized conversion rate of prediabetes to diabetes is estimated to be 5%–10% (Tabák et al., *Lancet*, 2012). Thus, it is important to show using MR that obesity is also a causal risk factor for the early stages of T2D development (i.e. prediabetes), which emphasizes the need to treat obesity as a causal factor from early on. 2) During the development of T2D, insulin secretion initially increases to compensate the insulin resistance and the disease occurs when β cell compensation fails. Thus, insulin resistance is a key marker for the development of T2D. However, due to the difficulty in accurately assessing insulin resistance in large populations, there are no prior MR analysis on insulin resistance. Our study on the unique METSIM cohort with an oral glucose tolerance test data available in ~10,000 individuals established the causal effect from obesity to insulin resistance, which pinpoints the key role of insulin resistance in the obesity induced prediabetes/diabetes. We have now revised the manuscript to elaborate these two aspects in our revised Introduction (page 4) and Discussion (page 18).

**“Next, we investigated the role of subcutaneous adipose tissue in these obesogenic effects. We discovered that the adipose mitochondrial gene expression and body mass index (BMI) are independently associated with insulin resistance after adjusting for the tissue heterogeneity.”**

**Here we have a problem as the text implies an interest to look at the role of subcutaneous adipose tissue, and the way they are doing that is in part looking into the associations with BMI. BMI is in no way a reliable indicator for subcutaneous adipose tissue. BMI would incorporate subcutaneous and all other types of adipose tissue, but it also reflects the amount of muscle mass. Indeed, it is known to have a strong gender bias, and muscular men are likely to be classified as ‘obese’ using BMI cut-offs (and even if their subcutaneous fat would be likely to be very limited). UK Biobank does have information on fat-percentage and other more refined indicators of adiposity, which could prodice helpful information in this context.**

**Related to the above and the overall conceptualisation in the paper. The authors use BMI interchangeably with ‘obesity’ but these are two very different issues. This is particularly concerning in the context of conclusions, where the they make mechanistic claims about the role of adipose tissue in obesity. Overall this study comes across as slightly odd mix of conducting analyses and conceptualisation in a very rough way (BMI=subcutaneous tissue=obesity) and yet aiming to get to more refined analyses and understanding through the cellular approaches.**

Response: We thank the Reviewer for the helpful suggestion. We agree that given that BMI cannot separate lean mass and fat mass (Ambrosi et al., *Obesity*, 2011), BMI is only a rough, though clinically most commonly used measure of obesity whereas fat percent is a more suitable trait to represent obesity. The Reviewer also made a good point about the fact that males and females have different fat/muscle distributions. Accordingly, we have now revised our approach and tested the fat percent in all our analyses of the METSIM and UK Biobank (UKB) cohorts. Our revised results using the fat percent instead of BMI for the MR analyses and Matsuda prediction model still support the same previous conclusions (pages 8-10 and pages 15-16 of the revised Results, revised Figure 1, and new Supplementary Fig. 4). Our new results also show that BMI and fat percent are highly correlated (new Supplementary Fig. 4); and that adipose MT expression is highly significantly associated with both BMI and fat percent in METSIM and with the obesity status in a new Mexican cohort, MOSS (new Supplementary Figure 1A-C). However, taken together we feel that the Reviewer’s suggestions greatly improved the reliability of our adipose tissue -related analyses by helping us to choose a better indicator of obesity. We have also thoroughly checked our entire manuscript to make sure that sex is always included as a covariate whenever applicable.

**Methods:**

**MR PRESSO does not control for pleiotropy, but it includes a test which can help to detect pleiotropy. In general, the advice is to include multiple different MR methods, including pleiotropy robust methods such as MR Eggr and others.**

Response: We thank the Reviewer for this helpful comment. While MR-PRESSO corrects for the potential pleiotropy and provides the corrected causal effects/p-values as a control for pleiotropy, we agree that additional methods are necessary for establishing the causal effects we described. Therefore, we also performed a heterogeneity test and MR egger test to further validate our MR results. These new results show that there is no evidence of heterogeneity or horizontal pleiotropy in our MR analyses. We have now revised our MR results accordingly (page 8-10, Figure 1).

**Instrument selection was based on the same dataset in which the effects were tested, which can introduce substantial bias into MR analysis (Burgess et al. 2016, Epidemiology). They investigators further filtered the BMI SNPs based on the observed associations in the METSIM cohort, potentially further aggravating the bias. I suggest the analyses to be revised such that the selection of instruments is based on an independent dataset, such as a signals from genomewide association meta-analyses on adiposity traits, assuming these do not include either of the cohorts used.**

Response: We thank the Reviewer for this helpful suggestion. For the MR analysis of obesity (fat percent) -> Matsuda index, we have now selected the IVs only based on the UKB GWAS results and do not filter IVs in the METSIM cohort anymore. Thus, the selection of IVs and the MR analyses are now performed in two independent cohorts. As for the bi-directional MR analysis of obesity (body fat percent) <-> prediabetes, we randomly separated the UKB cohort into two independents groups and performed a 2 sample MR analysis. These new analyses showed the same significant causal effects of obesity (body fat percent) -> Matsuda index (p-value = 3.1e-24) and obesity (fat percent) -> prediabetes (p-value = 1.2e-3) (page 8-10, figure 1) while MR-egger did not detect any significant pleiotropy (all P.pleio > 0.05).

**They claim that they confirmed that the instruments were not associated with any confounders, but there were no analyses to support this claim. It appears that the authors take this claim based on MR presso, which suggests some confusion about related methods.**

Response: To address this comment, we have now excluded the GWAS variants of diastolic/systolic blood pressure and total triglycerides from the MR analyses of obesity (body fat percent) <-> prediabetes since blood pressure and dyslipidemia are reported risk factors of T2D. In more detail, we first performed the GWAS analyses of diastolic/systolic blood pressure and total triglycerides in the UKB cohort and then excluded those GWAS variants from our MR analysis of obesity (represented by body fat percent) -> prediabetes. As it is not practical to identify and test for all possible cofounders though GWAS analyses, we also now performed a heterogeneity test and MR egger test to identify any possible signs of pleiotropy. We have revised the manuscript to clarify that we did not observe any evidence for pleiotropy based on the MR-PRESSO, MR egger, or heterogeneity test (page 8-10, Figure 1).
