# Supplementary material for: The causal effect of obesity on prediabetes and insulin resistance reveals the important role of adipose tissue in insulin resistance
Source: PLoS Genet. 2020 Sep 14;16(9):e1009018. doi: 10.1371/journal.pgen.1009018 (PMC7515203; doi:10.1371/journal.pgen.1009018)
Supplement: Attachment — Submitted filename: Reviewer2_final.docx [file pgen.1009018.s021.docx]

**Responses to Reviewer 2:**

We thank the Reviewer for the helpful critique and comments of the manuscript and have addressed all of the issues that were raised. We hope that the revisions satisfactorily respond to all of the Reviewer’s concerns. The changes are indicated in the marked-up copy of the revised manuscript.

**Please test the associations of the IV with confounders, e.g. sex, education, social class, physical activity, smoking, and alcohol. At the moment the paper infers lack of confounder associations from comparisons across MR approaches which is not appropriate.**

Response: We thank the Reviewer for this constructive comment. We have now tested for association between the IVs and physical activity, smoking, and alcohol intake. Only 4 IV variants showed a significant association with the tested confounders, and removing the 4 variants did not affect the results in any of the 3 MR analyses. We have now revised the manuscript to include these new results (page 10 and the new Supplementary figure 1).

However, to the best of our understanding the association test between IVs and potential confounders is not generally considered as a mandatory test for MR analyses. First, it is impractical to test for the associations between the IVs and all potential confounders. Second, even if associations were identified between the IVs and the confounders, the associations can be caused by multiple reasons, as illustrated below. Among the illustrated potential paths in the figure below, only the 5^th^ one indicates a horizontal pleiotropy, which violates the MR assumptions. Thus, when compared to the association test between the IVs and potential confounders, sensitivity tests, such as heterogenous Q test or MR-egger that we used, are more commonly applied for detecting pleiotropy in many previous MR analyses^[1]^^[2]^^[3]^.

**I would suggest a tidy up of terminology, and an aim to revise to simply refer to things for what they are. If a study has shown something is related to higher BMI, then why not simply refer to association between higher BMI and a given outcome, rather than labelling it ‘obesity’ (represented by BMI)? Obesity is when BMI is >30 (WHO classification), while in a continuous scale a higher BMI can occur across the whole range of BMIs.**

Response: We thank the Reviewer for this helpful comment. We have now used “high BMI” instead of “obesity” when we described the particular previous study (pages 6, 14). We also revised the manuscript to make sure that the terminology regarding this issue is consistent across all sections.

**If outliers are not detected, MR – presso produces estimates that are identical to IVW, which assumes no horizontal pleiotropy. The authors write that they used MR presso to “correct for pleiotropy and test for causal effects”. How was pleiotropy corrected? If there indeed was no outliers identified what did they correct for? I suggest removing statements relating to “correcting for pleiotropy”. It is good also to appreciate, that none of the MR methods are “perfect” and the absence of evidence for pleiotropy does not necessarily prove the absence of pleiotropy.**

Response: We thank the Reviewer for this constructive comment. Usually MR-PRESSO^[4]^ identifies the potential outliers and removes the outliers to correct for pleiotropy. However, we agree with the Reviewer that since MR-PRESSO did not identify any outliers, there is no pleiotropy to correct for. Thus, we revised the Methods section and removed the statements about correcting for pleiotropy (page 24), as suggested by the Reviewer. We also added the comment into the revised Method section to clearly state that no perfect method exists as of yet to guarantee a pleiotropy free MR analysis (page 24-25).

1. Bretherick, A. D. et al. Proteome-by-phenome Mendelian Randomisation detects 38 proteins with causal roles in human diseases and traits. bioRxiv 631747 (2019) doi:10.1101/631747.
2. Nowak, C. *et al.* Effect of Insulin Resistance on Monounsaturated Fatty Acid Levels: A Multi-cohort Non-targeted Metabolomics and Mendelian Randomization Study. *PLoS Genet.* **12**, 1–19 (2016).
3. Millard, L. A. C., Davies, N. M., Tilling, K., Gaunt, T. R. & Davey Smith, G. Searching for the causal effects of body mass index in over 300 000 participants in UK Biobank, using Mendelian randomization. PLoS Genet. 15, 1–20 (2019).
4. Verbanck, M., Chen, C. Y., Neale, B. & Do, R. Detection of widespread horizontal pleiotropy in causal relationships inferred from Mendelian randomization between complex traits and diseases. Nat. Genet. 50, 693–698 (2018).
